# Development of Hairy Root Cultures for Biomass and Triterpenoid Production in *Centella asiatica*

**DOI:** 10.3390/plants11020148

**Published:** 2022-01-06

**Authors:** Seungeun Baek, Jong-Eun Han, Thanh-Tam Ho, So-Young Park

**Affiliations:** 1 Department of Horticultural Sciences, Chungbuk National University, Cheongju 28644, Korea; bse618@hanmail.net (S.B.); year300@naver.com (J.-E.H.); 2 Hankook Cosmetics Manufacturing Co., Ltd., 20F, 35, Cheonggyecheon-ro, Jongno-gu, Seoul 30188, Korea; 3 Institute for Global Health Innovations, Duy Tan University, Da Nang 550000, Vietnam; 4 Faculty of Pharmacy, Duy Tan University, Da Nang 550000, Vietnam

**Keywords:** *Centella asiatica*, hairy root culture, medicinal plant, *rol* genes, triterpenoid

## Abstract

*Centella asiatica* (Apiaceae) is a tropical/subtropical medicinal plant, which contains a variety of triterpenoids, including madecassoside, asiaticoside, madecassic acid, and asiatic acid. In this study, we tested the efficiency of hairy root (HR) induction in *C. asiatica* from leaf and petiole explants. Leaves and petioles collected from *C. asiatica* plants were suspended in agro-stock for 30 min and co-cultured with *Agrobacterium rhizogenes* for 3 days to induce HR formation. The transformation efficiency of leaf and petiole explants was approximately 27% and 12%, respectively. A total of 36 HR lines were identified by PCR-based amplification of *rol* genes, and eight of these lines were selected for further analysis. Among all eight HR lines, the petiole-derived lines HP4 and HP2 displayed the highest growth index (37.8) and the highest triterpenoids concentration (46.57 mg∙g^−1^), respectively. Although triterpenoid concentration was >2-fold higher in leaves than in petioles of *C. asiatica* plants, the accumulation of triterpenoids in petiole-derived HR cultures was 1.4-fold higher than that in leaf-derived HR cultures. Additionally, in both leaf- and petiole-derived HR cultures, terpenoid production was higher in HRs than in adventitious roots. These results demonstrate that the triterpenoid content in the explant does not affect the triterpenoid content in the resultant HRs. The HR culture of *C. asiatica* could be scaled up to enable the mass production of triterpenoids in bioreactors for the pharmaceutical and cosmetic industries.

## 1. Introduction

*Centella asiatica* belongs to the Apiaceae family (Figure S1) and is a perennial, prostrate, herbaceous creeper plant. The stems are slender, with long stolons to connect plants to each other [1]. *C. asiatica* is a medicinal plant native to the tropical and subtropical countries of South and Southeast Asia as well as South Africa and Madagascar [2]. They have been used in traditional medical therapy, which showed that they had potential effects on numerous physical and mental ailments such as antitumor activity, immunomodulation, antidepressant activity, stomach pain, leucoderma, urinary discharges, inflammations, fevers, and syphilitic skin diseases, etc. [2,3]. Triterpenoids, including madecassoside, asiaticoside, madecassic acid, and asiatic acid (Figure S2), found in *C. asiatica* are responsible for its therapeutic effects as these substances exhibit memory enhancing activity, antimutagenic effects, and antioxidant activity (required for wound healing) and promote fibroblast production, collagen I synthesis, collagen III secretion, and angiogenesis [4,5,6]. In recent years, *C. asiatica* has been increasingly used in pharmaceutical and cosmetic industries worldwide [7,8,9]. However, the geographical distribution of *C. asiatica* is limited to the wetlands of tropical or subtropical areas, and the growth of this species is slow in natural environments. Moreover, the contents of bioactive compounds are high in leaves and low in other tissues [10]. For example, asiaticoside and madecassoside contents are low in the root, suspension cell culture, and callus of *C. asiatica* [10,11]. Therefore, alternative strategies, such as in vitro plant cell and tissue culture, are needed for the efficient production of bioactive compounds in *C. asiatica*.

Adventitious roots (ARs) and hairy roots (HRs) of many medicinal plants are recognized as suitable systems for the production of biomass and bioactive compounds [12]. HR cultures produced by plant transformation with the natural vector system, *Agrobacterium rhizogenes*, exhibit high-level secondary metabolite production. T-DNA, which carries a set of genes encoding auxin and cytokinin biosynthesis enzymes [13,14], is transferred from the Ri-plasmid of *A. rhizogenes* to the genome of the target plant. Thus, the resultant HR cultures exhibit relatively fast growth (in hormone-free media), high genetic and biochemical stability, and efficient bioactive compound synthesis [15,16]. The production of asiaticoside and madecassoside in *C. asiatica* in vitro and in vivo have been reported by Aziz et al. (2007) [17], of which the highest content was found in leaves. Unfortunately, both asiaticoside and madecassoside were undetectable in hairy roots and callus culture in this study [17]. Kim et al. (2007) also obtained approximately 14.1% hairy root from leaf and petiole in *C. asiatica*, and enhanced the production of asiaticoside in hairy root culture by subjecting it to 100 µM methyl jasmonate [11]. In addition, the comparison of the growth and triterpenoid production between the diploid and tetraploid in *C. asiatica* hairy root culture was observed, of which diploid hairy root produced higher triterpenoid (1.7-fold) than tetraploid hairy root [18]. Although HR cultures of *C. asiatica* have been generated from leaf, petiole, stolon, and callus tissues, enhancing the accumulation of triterpenoids and other secondary metabolites in these cultures has been difficult [11,17,18,19]. In the present study, we examined whether the explant type affects the terpenoid content of HR and AR cultures of *C. asiatica*. We also compared the growth and metabolite contents of HR and AR cultures to determine the most effective strategy for producing triterpenoids.

## 2. Results and Discussion

### 2.1. Induction and Characterization of HRs

**Figure 1:**
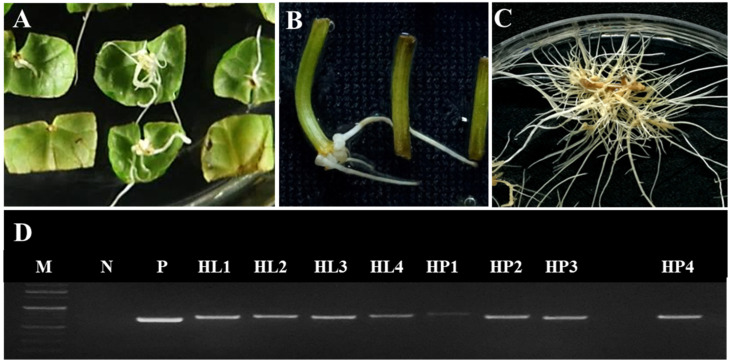
Induction of hairy roots (HRs) in *Centella asiatica* using *Agrobacterium rhizogenes* strain KCCM 11879 after 3 weeks of culture. (**A**,**B**) HR induction from leaf explants (**A**) and petiole explants (**B**); (**C**) HR proliferation; (**D**) PCR analysis of the rolB gene from HR lines. M, ladder; N, negative control (non-transformed root); P, positive control (*Agrobacterium rhizogenes*); HL1–4, HR lines derived from leaf explants; HP1–4, HR lines derived from petiole explants.

In *C. asiatica*, HRs were obtained from leaf and petiole explants obtained from in vitro-grown plantlets by co-cultivation with *A. rhizogenes* strain KCCM 11879. The explants were cultured on MS medium containing antibiotics. At 3 weeks, the transformation efficiency of petiole and leaf explants was approximately 12% and 27%, respectively (Table 1). In the leaf explant, multiple HRs were induced from main vein and most of them were directly induced from the explant (Figure 1). In previous studies, the highest HR induction rate was observed at the junction between the leaf and petiole tissues [11]. When plants are infected by *A. rhizogenes*, the auxin biosynthesis-related genes present in the T-DNA of the Ri-plasmid are integrated into the plant genome. Therefore, HRs can be induced without the addition of growth regulators, especially auxin [20]. The efficiency of HR induction has been associated with the source explant [21,22]. In *Valeriana sisymbriifolium*, the HR induction rate from leaf and petiole explants was higher than that from hypocotyls [22]. The high regeneration capacity of leaf explant in comparison with root, hypocotyl, and cotyledon explants has also been reported in other studies [23,24]. According to Hosoki and Asahira (1980) [25], intercalary meristems distributed in leaves are likely to be responsible for their high regeneration potential. Because the wounded area of the leaf was larger than the petiole size, we predicted that bacterial infection of the leaf explant would be easier than that of the petiole explant. Both explant types were subcultured on antibiotic-containing medium at weekly intervals, and the remaining bacteria were completely removed after 4 weeks of culture.

### 2.2. Confirmation of HR Transformation

To confirm the insertion of the 652 bp *rol*B gene into the plant genome, 40 putative HR lines were examined by PCR. The insertion of *rol*B gene was confirmed in a total of 36 HR lines, including 19 leaf-derived and 17 petiole-derived lines. No amplification was detected in non-transgenic ARs (negative control) (Figure 1D). These results indicated that the pRi T-DNA fragment of *A. rhizogenes* was successfully integrated into the *C. asiatica* genome. Of the transformed HR lines, four leaf-derived and four petiole-derived HR lines, which showed healthy growth and extensive branching, were selected for further analysis.

### 2.3. Growth and Proliferation of HR Lines

**Figure 2:**
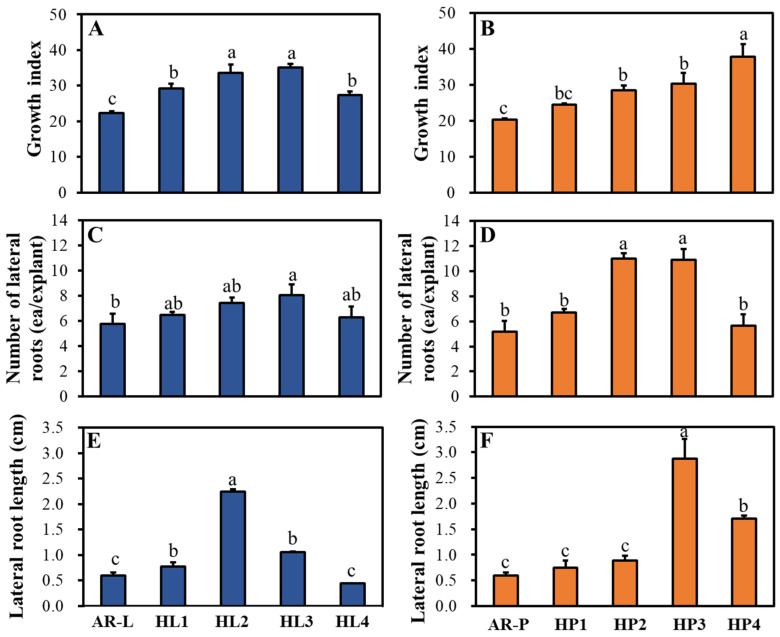
Growth characteristics of different HR lines after 3 weeks of culture. (**A**,**C**,**E**) Growth index (**A**), lateral root number (**C**), and lateral root length (**E**) of non-transgenic adventitious root (AR) and HR cultures induced from leaf. (**B**,**D**,**F**) Growth index (**B**), lateral root number (**D**), and lateral root length (**F**) of AR and HR cultures induced from petiole. AR-L and AR-P, leaf- and petiole-derived AR lines, respectively; HL and HP, leaf- and petiole-derived HR lines, respectively. Data represent mean ± standard error (SE). Different lowercase letters above bars indicate significant differences (Duncan’s multiple range test [DMRT]; *p* ≤ 0.05).

The HR lines showed rapid growth over the culture period. After culturing, the root tip explants continued to elongate and produced numerous branches. The growth rate of HR lines was higher than that of non-transgenic roots. HRs derived from petiole explants showed better growth than those derived from leaf explants (Figure 2A,B). The petiole-derived line HP4 showed the highest GI of 37.8. Additionally, comparison of the morphological characteristics of HR lines revealed that line HP3 showed the highest number and length of lateral roots (Figure 2C–F). Additionally, the number and length of lateral roots of HR lines were greater than those of AR lines derived from both leaf and petiole explants. The *rol*A, *rol*B, and *rol*C oncogenes of *A. rhizogenes* are known to control cell differentiation and growth in plants [26]. The insertion of the T-DNA containing the *rol* genes into the plant genome leads to the induction of HR formation without the exogenous application of plant growth regulators. The *A. rhizogenesis* strain KCCM 11879 has been successfully integrated into the *Polygonum multiflorum* genome using leaf explants, resulting in the generation of an HR line with high biomass and phenolic compound content [12].

### 2.4. Comparison of Triterpenoid Accumulation between HR Lines and AR Cultures

**Figure 3:**
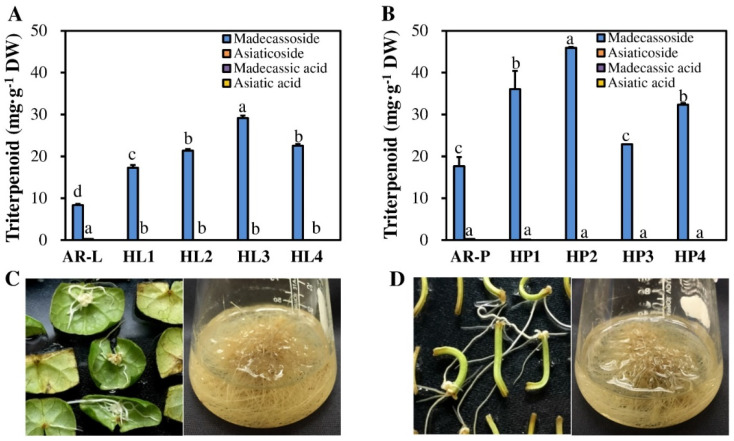
High performance liquid chromatography (HPLC) analysis of triterpenoids in the adventitious root (AR) and hairy root (HR) lines of *C. asiatica*. (**A**) Triterpenoid contents of AR (AR-L) and HR (HL1-HL4) lines derived from leaves. (**B**) Triterpenoid contents of AR (AR-P) and HR (HP1-HP4) lines derived from petioles. (**C**,**D**) Photographs showing HR induction from leaves (**C**) and petioles (**D**) as well as HR proliferation. Data represent mean ± SE. Different lowercase letters above bars indicate significant differences (DMRT; *p* ≤ 0.05).

**Figure 4:**
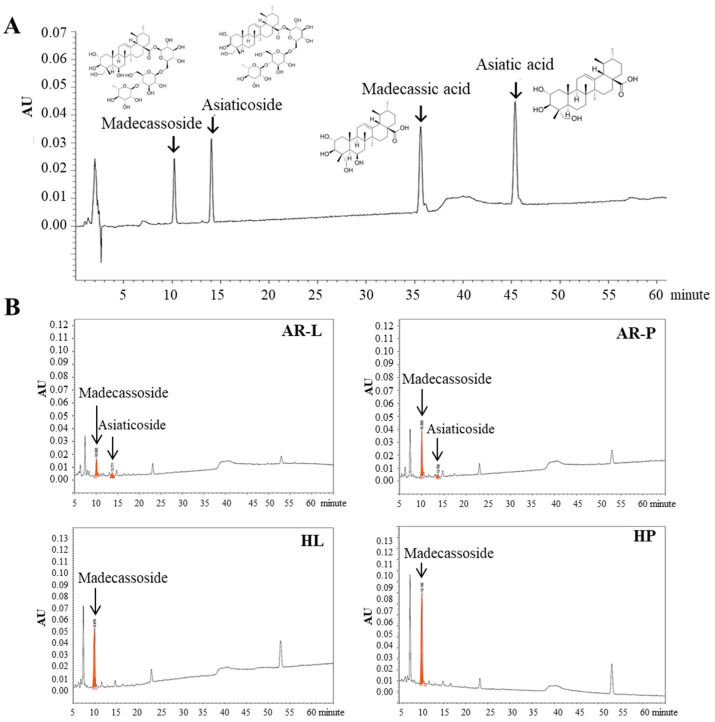
HPLC chromatograms of triterpenoids. (**A**) Standard triterpenoids. (**B**) Triterpenoids identified in the AR and HR lines derived from different explant types. AR-L, -P: AR lines derived from leaf and petiole explants, respectively; HL and HP: HR lines derived from leaf and petiole explants, respectively.

HR cultures are attractive alternative sources of plant bioactive compounds as they are genetically stable, exhibit high biomass production, and possess a biosynthetic capacity comparable with that of the natural plant root [14]. In this study, the terpenoid contents of eight leaf- and petiole-derived HR lines and non-transgenic leaf- and petiole-derived ARs were analyzed by HPLC (Figure 3 and Figure 4). All HR lines showed greater accumulation of triterpenoids than the non-transgenic ARs. When compared with the other transformed lines and non-transgenic roots, the petiole-derived HR lines showed higher levels of total triterpenoids than leaf-derived HR lines and non-transgenic ARs. Among them, the HP2 line showed the highest total triterpenoid content (46.57 mg∙g^−1^ dry weight [DW]), which was 1.6-fold higher than line HL3 (the highest leaf-derive HR line), and 2.6-fold higher than that of petiole-derived ARs (AR-P) (Figure 3 and Figure 4). While madecassoside was detected in all AR and HR lines, asiaticoside was found only in leaf- and petiole-derived ARs. During the genetic transformation process, the T-DNA region of the Ri-plasmid of *A. rhizogenes* harboring the *rol*A–C genes is integrated into the plant genome, thus inducing the formation of HRs and affecting the production of bioactive compounds [27,28]. The role of *rol* genes in the production of secondary metabolites has been widely reported [14,26]. For example, *rol*B resulted in the overproduction of resveratrol in *Vitis amurensis* [29]; *rol*A stimulated the production of nicotine [30]; *rol*C enhanced the tropane alkaloid content of HRs in *Atropa belladonna* [31].

In addition, secondary metabolites, including terpenoids, alkaloids, and phenolics, often accumulate to higher levels in HR cultures than in cell or callus cultures [14,32]. In this study, almost all HRs lines (except line HP3) showed higher triterpenoid contents than the non-transformed ARs. This result is consistent with the study of Ho et al. (2018) [12], who showed that the phenolic content of HRs is higher than that of ARs in *P. multiflorum*. Thus, HR cultures of *C. asiatica* are potentially a valuable source of secondary metabolites, and line HP2 is optimal for biomass and triterpenoids production.

### 2.5. Comparison of Terpenoid Accumulation among HR Lines, AR Cultures, and Source Explants

**Figure 5:**
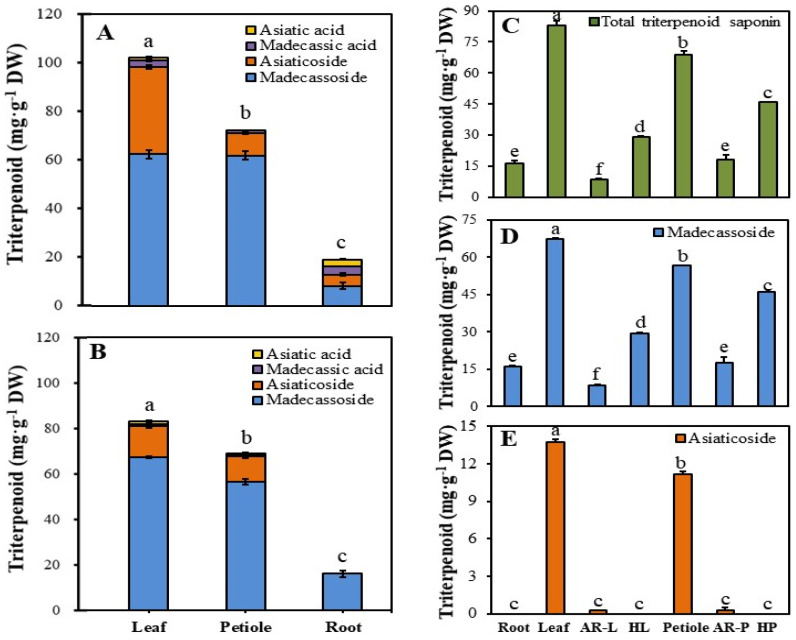
Comparison of the triterpenoid contents in HR lines, ARs, and source explants. (**A**) Ex vitro-grown plant; (**B**) In vitro-grown plant; (**C**) total triterpenoid contents, (**D**) madecassoside content; (**E**) asiaticoside content. AR-L and -P, leaf- and petiole-derived AR lines, respectively; HL and HP, leaf- and petiole-derived HR lines, respectively. Data represent mean ± SE. Different lowercase letters above bars indicate significant differences (DMRT; *p* ≤ 0.05).

Next, we determined the triterpenoid contents in different organs (leaf, petiole, and root) of ex vitro- and in vitro-grown plants by HPLC analysis. Among all the samples tested, triterpenoid contents were the highest in the leaves of ex vitro- and in vitro-grown plants, and lowest in the roots of ex vitro- and in vitro-grown plants (Figure 5A,B). Madecassoside was highly abundant in all plant parts. In ex vitro plants, the content in asiaticoside was the highest in leaves (35.98 mg∙g^−1^ DW), but only 25% of its content in leaves was found in petioles (9.04 mg∙g^−1^ DW). On the other hand, in in vitro plants, the asiaticoside content in leaves was 1.2-fold higher than that of the petioles (constituting 55.2% and 44.8% of the total triterpenoid, respectively) but was absent in roots. These results were partially similar to those of previous studies. For example, Kim et al. (2004) reported that asiaticoside formed 82.6%, 15.9%, and 1.5% of the total triterpenoid content in leaves, petioles, and roots, respectively [10]. Thus, based on the results of the current study and previous studies, it can be postulated that asiaticoside exhibits tissue-specific accumulation in the aerial plant parts. Unlike madecassoside and asiaticoside, both asiatic acid and madecassic acid were detected in small quantities in ex vitro- and in vitro-grown plants (Figure 5A,B). From these results, it was reasonable to induce HRs from leaf and petiole, which had highest triterpenoid content.

To confirm the relationship between HRs and its source tissues, the total triterpenoid, madecassoside, and asiaticoside contents of the different plantlet parts, HRs, and ARs were analyzed (Figure 5C–E). Because the proportion of madecassoside in triterpenoid was the highest, the total triterpenoid and madecassoside contents showed a similar trend. Madecassoside content was high in the aerial parts of *C. asiatica* plantlets, with the highest value in leaves, which was 1.2-fold higher than the value in petioles. However, the ARs and HRs derived from leaf and petiole explants showed an opposite trend to that of the leaves and petioles of *C. asiatica* plantlets. Asiaticoside showed the highest content in leaves (13.76 mg∙g^−1^ DW). A small amount of asiaticoside (0.26 mg∙g^−1^ DW) was detected in ARs derived from leaves and petioles, but no asiaticoside was detected in HRs and plant roots. The content of secondary metabolites varies among the different plant parts. One-year-old *Panax ginseng* plants showed the highest content of ginsenosides in leaves and insufficient content in roots [33]. In soybean, flavanones were detected in leaves and stems but not in roots [34]. In the present study, four kinds of triterpenoids were detected in the aerial parts of in vitro-grown *C. asiatica* plantlets; however, only madecassoside was detected in roots.

**Figure 6:**
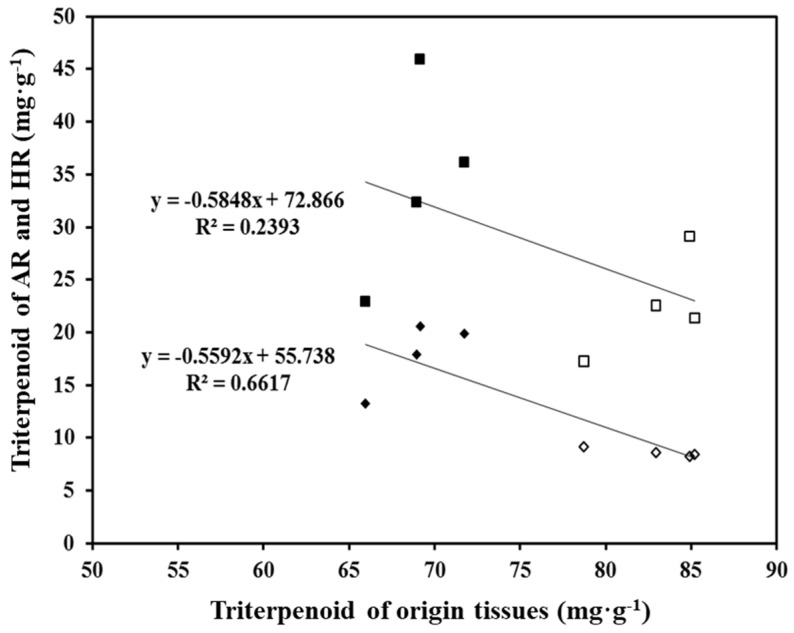
Correlation between the triterpenoid content in origin explants and the resultant HR and AR lines after 3 weeks of culture. ■, petiole-derived HR lines; □, leaf-derived HR lines; ◆, petiole-derived AR lines; ◇, leaf-derived AR lines.

Next, we compared the triterpenoid contents in HRs, ARs, and their source explant (leaf and petiole). The content of triterpenoid in leaf explants (84.35 mg∙g^−1^ DW) was 1.2-fold higher than that in petiole explants. On the other hand, the triterpenoid content in petiole-derived ARs (17.9 mg∙g^−1^ DW) was 2.0-fold higher than that of leaf-derived ARs. A similar trend was observed in HRs: the triterpenoid content in petiole-derived HRs was 1.4-fold higher than that of leaf-derived HRs. This confirmed that the triterpenoid contents in HRs and their source explants are negatively correlated. Thus, the triterpenoid content in the source tissue does not affect the triterpenoid content in the derived HRs (Figure 6). These results suggest that the ability of *C. asiatica* HR cultures to rapidly accumulate biomass is important for producing triterpenoids for industrial applications, such as for producing cosmetics and pharmaceuticals.

## 3. Materials and Methods

### 3.1. Plant Materials

In vitro-grown *Centella asiatica* (L.) Urb. was used in this study. To perform *Agrobacterium*-mediated transformation, shoot tips were surface-sterilized with 70% (*v*/*v*) ethanol for 30 s, followed by 2% (*v*/*v*) sodium hypochlorite for 10 min, and then rinsed three times with sterile distilled water. The sterilized shoot tips were placed on solid Murashige and Skoog (MS) medium [35], which was supplemented with 30 g∙L^−1^ sucrose and 2.4 g∙L^−1^ gelrite, adjusted to pH 5.8 (before adding gelrite), and then autoclaved at 121 °C for 15 min. The plates containing shoot tips were incubated at 24 ± 1 °C under a 16 h light/8 h dark photoperiod. The plantlets were subcultured every 6 weeks and used for subsequent experiments.

### 3.2. HR Induction

*Agrobacterium rhizogenes* strain KCCM 11879, obtained from the Korean Culture Center of Microorganisms (KCCM), Korea, was used for the induction of HRs. Briefly, the bacteria were grown on solid Nutrient Agar (NA) medium for 48 h. A single colony was used to inoculate 20 mL of liquid NA medium and grown overnight at 28 °C. Then, 2 mL of the inoculated NA medium was used to inoculate MS medium containing 100 μM acetosyringone, and the culture was grown for 24 h at 28 °C. The optical density of the *Agrobacterium* culture was measured at an absorbance of 600 nm (OD600) and adjusted to 1.0. Then, wounded leaf (1.0 cm × 1.5 cm) and petiole (1 cm in length) explants obtained from in vitro-grown plants were co-cultured with 20 mL of the *Agrobacterium* culture for at least 30 min at 28 °C. The explants were then removed from the bacterial suspension, placed on top of a dry sterilized filter paper to remove excess bacteria, and co-cultivated on MS medium supplemented with 30 g∙L^−1^ sucrose and 2.4 g∙L^−1^ gelrite for 3 days in the dark. After 3 days, explants were transferred to MS medium supplemented with 30 g∙L^−1^ sucrose, 2.4 g∙L^−1^ gelrite, 50 mg∙L^−1^ carbenicillin, and 25 mg∙L^−1^ cefotaxime to remove excess bacteria. The explants were incubated at 24 ± 1 °C in the dark for a total of 4 weeks, with subculturing every week using antibiotic-containing fresh medium. The hairy roots were maintained in a 100 mL flask containing 40 mL liquid MS medium supplemented with 50 g∙L^−1^ sucrose. On the other hand, the adventitious roots were induced from the leaf and petiole in MS medium supplemented with 1.0 mg·L^−1^ IBA, 30 g·L^−1^ sucrose, and maintained in a 100-mL flask containing 40 mL liquid MS medium with the same condition above. Both the hairy root and adventitious root cultures were incubated in the dark at 24 ± 1°C in a shaker at 100 rpm and subcultured every 3-week of culture for further study.

### 3.3. Genomic DNA Extraction and PCR Analysis

Genomic DNA was extracted from the non-transformed ARs and HRs using the cetyl trimethyl ammonium bromide (CTAB) method [36], with slight modifications. DNA was dissolved in TE buffer. The *rol*B gene was amplified from the T-DNA of *A. rhizogenes* strain KCCM 11879 (positive control) and the genomic DNA of non-transformed ARs and HRs by PCR (Gnc Bio Co., Ltd., Korea) using gene-specific primers (forward, 5′-ACTATAGCAAACCCCTCCTGC-3′; reverse, 5′-TTCAGGTTTACTGCAGCAGGC-3′) under the following conditions: initial denaturation at 95 °C for 5 min, followed by 30 cycles of denaturation at 95 °C for 5 min, annealing at 58 °C for 30 s, and extension at 72 °C for 45 s, and a final extension step at 72 °C for 5 min. To detect the expected amplicon of 652 bp, the PCR products were analyzed by electrophoresis on a 2% (w/v) agarose gel.

### 3.4. Characterization of HR Lines

A total of eight HR lines, including four derived from leaves (HL1–4) and four from petioles (HP1–4), were placed on MS medium and cultured for 3 weeks. The number and length of lateral roots of each HR line were determined after 2 weeks, and growth index (GI) was calculated using the following equation

Growth index (GI) = (Harvested FW − Inoculated FW)/Inoculated FW(1)

FW (fresh weight) represents the fresh weight of the HR line.

Four triterpenoids were analyzed in each of the eight lines by high performance liquid chromatography (HPLC), and the capacity of each line to produce secondary metabolites was also determined. To determine the relationship between each HR line and its source explant, their triterpenoid contents, determined using HPLC, were compared.

### 3.5. Analysis of Triterpenoids by HPLC

Authentic HPLC-grade (purity > 98%) triterpenoids (madecassoside, asiaticoside, madecassic acid, and asiatic acid) were obtained from ChromaDex (Laguna Hills, CA, USA). HRs (200 mg dry weight) were ground in a TissueLyser (QIAGEN, Hilden, Germany) and extracted with 4 mL of methanol by 2 h sonication (MUJIGAE Co., Ltd., Seoul, Korea). The extracts were centrifuged at 2000 rpm for 20 min, and the supernatant of each sample was transferred to a clean tube. The supernatant of each sample was diluted 20-fold and filtered through a membrane filter (pore size, 0.45 μm; Millipore). The diluted supernatants were analyzed by HPLC using the Waters 2690 Separations Module (Milford, Ma, USA) and Waters 996 photodiode array (PDA) detector. Separation was primarily achieved using a C18 column (4.0 mm × 250 mm; i.d., 5.0 μm) with acetonitrile (A) and 0.3% aqueous phosphoric acid (*v*/*v*) (B) as the mobile phase. Aliquots of 20 μL were injected into the HPLC system, and samples were eluted at a constant flow rate of 1.0 mL∙min^−1^ using the following gradient program: 22% A and 78% B for 0–65 min; 55% A and 45% B for 65–66 min; 95% A and 5% B for 66–76 min; 22% A and 78% B for 76–86 min. Calibration plots were obtained by measuring the peak areas at 200 nm wavelength. UV absorption spectra and retention times were used as criteria for the identification of 4 major compounds.

### 3.6. Statistical Analysis

The results are presented as the mean values of this experiment. One-way analysis of variance (ANOVA) was used to determine whether the differences among groups were statistically significant. Then, pairwise comparisons between means were assessed using Duncan’s multiple range test. A *p*-value of 0.05 was considered to indicate statistical significance. All statistical analyses were performed using the SAS program (version 9.4; SAS Institute Inc., Cary, NC, USA).

## 4. Conclusions

The present study showed that hairy roots were an effective system to produce triterpenoid from *C. asiatica,* and the results indicated that the content in the source tissue does not affect the triterpenoid content in the HRs and ARs of *C. asiatica*. Petiole explants proved a better source for inducing hairy roots and triterpenoid production than leaf explants. The petiole-derived HR line HP2 showed the highest-level accumulation of triterpenoids, and higher than 2.6-fold when compared to adventitious root culture. The HR culture of *C. asiatica* could be scaled up to enable cultivation in bioreactors, thus providing a profitable and long-lasting source of triterpenoids for the pharmaceutical and cosmetic industries.

## Figures and Tables

**Table 1:** Induction of hairy roots (HRs) from petiole and leaf explants of *Centella asiatica* using a 3-week-old culture of *Agrobacterium rhizogenes* strain KCCM11879.

Explant	Treatment	No. of Explants	Survival Rate (%)	Frequency of Root Formation (%)	No. of Confirmed HR ^z^ Lines
Leaf	Control	50	100 ± 0.0 a ^y^	4.0 ± 0.1 b	0.0 ± 0.0 b
	KCCM 11879	250	100 ± 0.0 a	26.8 ± 2.7 a	19.1 ± 0.7 a
Petiole	Control	90	100 ± 0.0 a	0.0 ± 0.1 b	0.0 ± 0.0 b
	KCCM 11879	450	100 ± 0.0 a	12.0 ± 1.6 a	17.0 ± 1.9 a

^z^ Confirmed hairy root by *rol* gene detection using PCR; ^y^ Different letters indicate significant differences at *p* < 0.05 according to Duncan’s multiple range test.

## Data Availability

All data is comprised in the manuscript.

## Supplementary Materials

The following supporting information can be downloaded at: https://www.mdpi.com/article/10.3390/plants11020148/s1, Figure S1: *Centella asiatica* characteristic. (A) field-grown plant; (B) leaf; (C) petiole; (D) root; Figure S2: Chemical structure of triterpenoids in *Centella asiatica*.
