# Supplementary material for: Development of Hairy Root Cultures for Biomass and Triterpenoid Production in Centella asiatica
Source: Plants (Basel). 2022 Jan 6;11(2):148. doi: 10.3390/plants11020148 (PMC8781555; doi:10.3390/plants11020148)
Supplement: Supplementary file 1 [file plants-11-00148-s001.zip › plants-1493699-supplementary.pdf]

plants

Supplementary Material

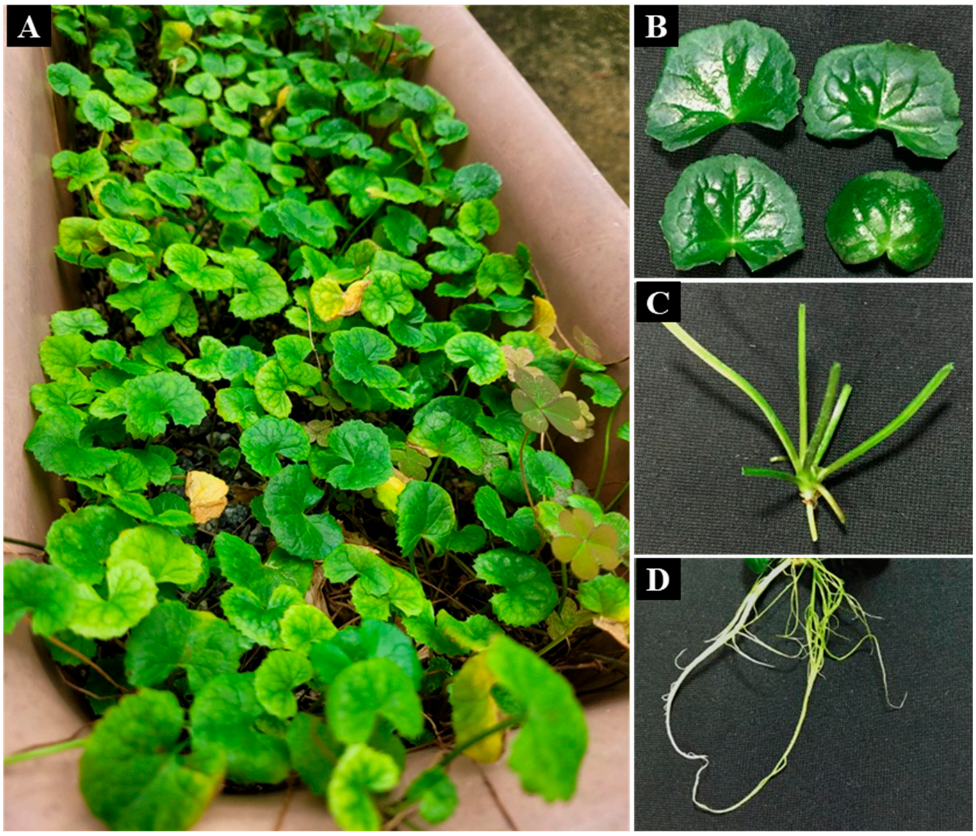

Figure S1. *Centella asiatica* characteristic. (**A**) field-grown plant; (**B**) leaf; (**C**) petiole; (**D**) root.

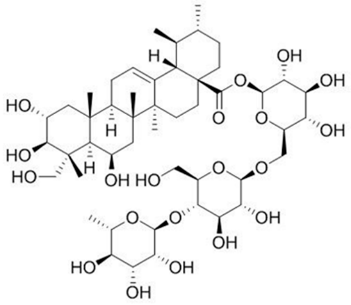

Madecassoside

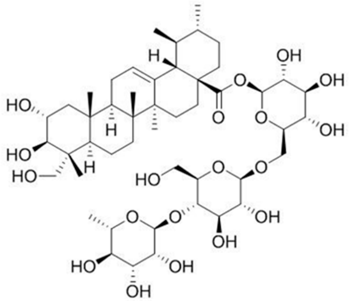

Asiaticoside

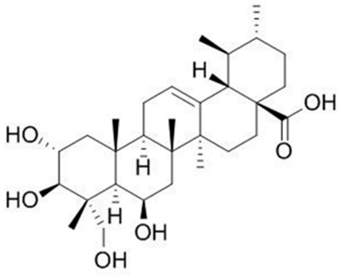

Madecassic acid

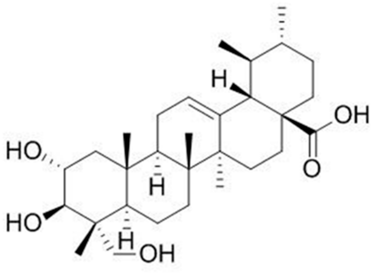

Asiatic acid

Figure S2. Chemical structure of triterpenoids in *Centella asiatica*.
